# Feasibility of Intensive Multidisciplinary Telerehabilitation Combined with Health Coaching for Underserved Stroke Survivors: A Pilot Study

**DOI:** 10.1177/26924366251383807

**Published:** 2025-10-03

**Authors:** Emily A. Stevens, Robert Suchting, Jane A. Anderson, Barbara Kimmel, Anette Ovalle, Anette Walder, Chethan P. Venkatasubba Rao, Neha Muraly, Carolyn P. Da Silva, Aylen Sosa, Allyson Seals Richard, Salvador Cruz-Flores, Mark P. Goldberg, DaiWai Olson, Ashley Zapata, Mary E. Russell, Sean I. Savitz

**Affiliations:** ^1^ Institute for Stroke and Cerebrovascular Disease, The University of Texas Health Science Center at Houston, Houston, Texas, USA.; ^2^ McGovern Medical School, The University of Texas Health Science Center at Houston, Houston, Texas, USA.; ^3^ Faillace Department of Psychiatry and Behavioral Sciences, McGovern Medical School at UTHealth Houston, The University of Texas Health Science Center at Houston, Houston, Texas, USA.; ^4^ Department of Neurology, Baylor College of Medicine and Center for Innovations in Quality, Effectiveness, and Safety (IQuESt), Houston, Texas, USA.; ^5^ Michael E. DeBakey Veterans Medical Center, Houston, Texas, USA.; ^6^ Department of Neurology, Baylor College of Medicine, Houston, Texas, USA.; ^7^ School of Physical Therapy, Texas Woman’s University, Houston, Texas, USA.; ^8^ Department of Physical Medicine and Rehabilitation, The University of Texas Health Science Center at Houston, Houston, Texas, USA.; ^9^ Department of Neurology, Texas Tech University Health Sciences Center El Paso, El Paso, Texas, USA.; ^10^ Department of Neurology and Medicine, UT Health Science Center at San Antonio, San Antonio, Texas, USA.; ^11^ Neuroscience Nursing Research Center, The University of Texas Southwestern Medical Center, Dallas, Texas, USA.

**Keywords:** stroke rehabilitation, stroke recovery, stroke, telerehabilitation, rehabilitation access, underserved stroke

## Abstract

**Introduction:**

Telerehabilitation (TR) can be as effective as in-clinic therapy; however, the implementation of barriers and facilitators to TR is unknown, especially in the underserved and rural population. In addition to TR, self-management support (SMS) interventions have been successful in improving outcomes for stroke survivors using telehealth. We explored the following: *(1) Is an intensive multidisciplinary TR intervention combined with SMS feasible to deliver virtual postacute stroke care? (2) Does an intensive TR intervention combined with SMS lead to improvements in level of impairment, functional outcomes, and quality of life? (3) Does an intensive TR intervention combined with SMS impact patient goal attainment? (4) What barriers and facilitators to TR are perceived by stroke survivors?*

**Methods:**

Virtually assisted home rehabilitation after acute stroke-2 offered two sessions of rehabilitation therapy, 3 days a week, for 4 weeks, consisting of two of the following disciplines: occupational therapy, physical therapy, or speech therapy. SMS was offered during the first and last session each week. Quantitative outcomes were completed at baseline assessment (week 1), postintervention assessment (week 6), and final assessment (week 10). Following grounded theory, semi-structured qualitative interviews were completed to identify barriers and facilitators of TR.

**Results:**

A total of *N* = 15 participants were consented into the program. When excluding the 3 participants who withdrew within or before week 1 of intervention, the average weekly number of therapy sessions completed by the remaining 12 participants was 5.6 (standard deviation [SD] 0.79), 5.6 (SD 0.90), 5.2 (SD 2.19), and 4.9 (SD 1.98) for weeks 2–5, respectively. Posterior probability (PP) results indicated very strong (PP >97%) to extreme (PP >99%) support in favor of change over time across most outcomes, including decreased modified Rankin Scale (marginal improvement of −0.731) and Patient Health Questionnaire scores (−3.606) and increased Montreal Cognitive Assessment (+4.178). Nine participants took part in the semi-structured interviews, and two major themes emerged: 1—“Perceived Access/Delivery” and 2—“Perceived Therapy Advantages.” In regard to the goal attainment, low sample sizes limited precision for analyses, thus these were not included in analyses.

**Conclusion:**

TR after acute stroke is feasible, though barriers still exist. This study proved to be a safe and attainable option for underserved populations of stroke survivors, demonstrating high attendance, improved outcomes, and no intervention-related adverse events.

## Introduction

Recent evidence suggests that despite decades-long improvements, stroke remains the third leading cause of death worldwide (7.3 million deaths; 10.7% of all deaths).^1^ Stroke is also the fourth most common cause of disability-adjusted life-years (DALYs; 160.5 million DALYs; 5.6%). Previous studies have shown that stroke recovery is fraught with challenges. In particular, survivors encounter difficulties in participating in follow-up stroke rehabilitation programs due to lack of transportation to rehabilitation centers, unsuitable scheduling, and insurance challenges.

Telerehabilitation (TR) has emerged as a valuable tool for health care professionals to reach patients who face challenges with transportation, reliance on caregivers, or who reside in rural areas. TR can address health disparities by expanding health care services to underserved populations. The provision of TR has allowed stroke survivors to receive therapy in high doses in their homes, with comparable or better results than traditional therapy.^2,3^ Research shows that incorporating tele-supervised rehabilitation via a TR system is effective in improving motor function, patient satisfaction, and quality-of-life outcomes in stroke survivors with hemiparesis and could ease the burden of caregivers.^4^ TR gives survivors an opportunity to accomplish their goals by increasing access and compliance with higher repetitions.^2^ Studies have shown that the dose of therapy when adding TR more than doubled what was provided by usual care, making it effective in the home environment.^2^ Moreover, TR can be utilized as a standalone or adjunct to traditional therapy, facilitating ongoing support and care tailored to the needs of stroke survivors, and leading to a better long-term recovery and overall reduction in disparities within the stroke community.^3^

In addition to TR, self-management support (SMS) interventions have been implemented in the stroke rehabilitation process to help stroke survivors face the challenges of stroke recovery and secondary prevention.^5–9^ SMS has been successful in improving health-related outcomes for survivors using telehealth,^5^ with digital health technologies further improving physical outcomes.^10^ Stroke survivors who completed SMS interventions via TR showed significant improvements in perceived disability and overall quality of life.^5^

We believe that there is a knowledge gap, as no previous studies have addressed the feasibility of intensive multidisciplinary intervention combining TR with health coaching for underserved stroke survivors. This is particularly important in the United States, as Texas has one of the largest rural populations with the most uninsured Americans.^11,12^ Building upon our prior experience and to close the knowledge gap, in this study, we explored the following questions: *(1) Is an intensive multidisciplinary TR intervention combined with SMS feasible to deliver virtual postacute stroke care? (2) Does an intensive TR intervention combined with SMS lead to improvements in level of impairment, functional outcomes, and quality of life? (3) Does an intensive TR intervention combined with SMS impact patient goal attainment? (4) What barriers and facilitators to TR are perceived by stroke survivors?*

## Methods

Virtually assisted home rehabilitation after acute stroke-2 (VAST-rehab 2) was a single-arm, feasibility (quantitative and qualitative) study of 15 patients implemented under the Lone Star Stroke Network.^13^ Institutional Review Board approval was granted by the Committee for Protection of Human Subjects at The University of Texas Health Science Center, Institutional Review Board for Baylor College of Medicine and Affiliated Hospitals, and Institutional Review Board at Texas Tech Health Science Center El Paso. This study was registered on clinicaltrials.gov (NCT05737524).

### Participants and recruitment

Participants were approached while hospitalized in acute care at Memorial Hermann—Texas Medical Center in Houston, Texas, Baylor College of Medicine and affiliated hospitals, or Texas Tech Health Science Center El Paso and affiliated hospitals. Inclusion criteria included: diagnosed with hemorrhagic or ischemic stroke, prestroke modified Rankin Scale (mRS) greater than 3, able to begin study activities within 3 months of symptom onset, age 18 or older, recommended to participate in rehabilitation therapies by a physician or rehabilitation therapist, sufficient cognitive and language abilities to comprehend verbal commands and carry out the study activities, able to access the internet, and mild to moderate impairment in motor or cognitive function. Exclusion criteria included: history of neurological or other disease resulting in significant functional impairment at baseline, diagnosed with a severe comorbid disorder that has a survival of less than 6 months, or any other condition that would preclude safe or effective participation in study activities.

Eligible and agreeable participants were consented either in person or remotely via informed consent. If consented remotely, electronic consent was completed via REDCap. Study activities were completed over video call via Health Insurance Portability and Accountability Act compliant UTHealth Webex or Zoom platforms. If participants experienced difficulty accessing the video call platforms, troubleshooting occurred with study staff in the participant’s preferred language (English or Spanish), and written/pictorial instructions were provided if needed.

### TR program

Participants received 2 hours of rehabilitation therapies per day, 3 days a week, for 4 weeks. Rehabilitation therapy interventions were based on participants’ individual needs and consisted of two of the following disciplines: occupational therapy, physical therapy, or speech therapy. Participants self-reported goals at the time of their baseline assessment. The therapies provided individualized interventions based on their recovery goals, as is completed in standard-of-care therapies, with a focus on neurorecovery, exercise, balance, gait, coordination, communication, and language.

### Outcome measures

Quantitative outcomes were assessed at week 1 (baseline assessment), week 6 (postintervention assessment), and week 10 (final assessment). The Stroke Impact Scale (SIS) is a self-report measure with well-established psychometric properties that assesses the following domains on a scale of 0–100: (1) strength, (2) hand function, (3) activities of daily living (ADLs), (4) mobility, (5) communication, (6) emotion, (7) memory and thinking, and (8) participation.^14–18^ The Short Form 12 (SF-12) is a health-related quality-of-life patient report measure, identifying both a mental and physical score, with higher scores indicative of better functioning.^19^ The Patient Health Questionnaire (PHQ-8) is a patient report measure of depressive symptoms, with a score equal to or greater than 10 indicative of depression.^20^ The mRS is a scale of global disability, with a lower score indicating less disability.^21^ The Montreal Cognitive Assessment (MoCA) is a screen for mild cognitive impairment with scores ranging up to 30, with normal at or above 26.^22^ The Goal Attainment Measure—Stroke assesses multiple aspects of goal attainment, with scores ranging from 0 to 8 and higher scores indicating goal attainment.^23^

### Qualitative interviews

Participant experiences were explored to identify barriers to and facilitators of TR intervention through in-depth interviews that occurred between weeks 6 and 10. Interviews were conducted in the participants’ preferred language (English or Spanish) to remove any language bias.

### Data analysis

#### Part 1: Quantitative data analytic strategy

Longitudinal change over time across each quantitative outcome was assessed via generalized linear mixed modeling (GLMM), a statistical modeling procedure that handles non-normally distributed outcomes via link functions and correlated observations via multilevel (“random” or “mixed”) effects.^24^ This method also allows for missing outcome data and/or data captured at unequal intervals. Each statistical model included one outcome regressed on time (measured in weeks) and a random intercept to account for repeated measures. To handle the bounded nature of each outcome’s distribution, values were converted to percentages of the maximum possible score and modeled via the Beta distribution. Outcomes that demonstrated values at the boundaries (0 or 1) were modeled via ordered Beta regression.^25^ The average marginal effect of time was calculated to provide the average increase or decrease in each outcome per one unit (week) change in the predictor (time); this value was then multiplied by 10 to describe the overall change over time from baseline to follow-up.

Bayesian statistical inference was used to quantify the probability that model effects exist, given the data and weakly informative priors (*b* ∼ *N* [*µ* = 0; *σ*^2^ = 1]). Bayesian inference was chosen for its accessible interpretation of probability (directly evaluating the alternative hypothesis) and favorable properties with small sample sizes.^26^ Model assumptions were tested via scale convergence factors (“rhat”), effective sample size, and posterior predictive checking. Posterior medians and 95% credible intervals (CrI) were taken as the point estimate and certainty range, respectively, for each regression coefficient. The proportion of the posterior distribution that was greater or less than the null effect (*b* = 0) was calculated as the posterior probability (PP) that the effect exists (i.e., PP[*b* >0 | *b* < 0]). For example, a 90% chance that the effect exists in the positive direction would be transcribed as PP(*b* >0) = 90%, and also imply a 10% chance that an effect exists in the other direction. The PP may be reconfigured to a Bayes factor (BF): the ratio of evidence in one direction or the other (e.g., 90%/10% = BF = 9.0). For practical application, heuristics^27–29^ characterize thresholds of evidence as *none* (PP = 50%), *anecdotal* (PP = 51–74%), *moderate* (PP = 75–90%), *strong* (PP = 91–96%), *very strong* (PP = 97–99%), and *extreme* (PP >99%). For the current analyses, the lower limit of the *moderate* threshold (75%) was set as the minimum level supporting the existence of model effects. Analyses were performed using *brms*,^30^ *ordbetareg*,^31^ and *marginaleffects*^32^ libraries in R.^33^

#### Part 2: Qualitative data analysis

Descriptive analysis of the participants’ sample was performed and is presented in the Results section (Table 4). Using a semi-structured interview guide, we obtained feedback from participants via Zoom, audio-recorded and transcribed interviews verbatim by the independent party. Data analysis was performed using the grounded theory approach to allow theory to emerge directly from the data.^34^ This process involved multiple steps of data collection, refinement, and interrelationship of categories of information. Two independent reviewers conducted thematic analysis by reading the transcripts, developed coding using the iterative consensus-building process, verified the codes, developed concepts, and created categories. A constant comparison process was performed to find emerging themes.

## Results

### Quantitative

A total of *N* = 15 participants were consented and enrolled into the program. Sample characteristics are provided in Table 1.

**Table 1.** Sample Characteristics

Characteristic	Mean	SD
Age	57.53	12.3
Days since stroke	60.93	52.18
NIHSS (at hospital admission)	9.87	7.96
NIHSS severity		
<5 (minor)	3	20.0%
5–15 (moderate)	9	60.0%
16–20 (moderate to severe)	1	6.7%
21–42 (severe)	2	13.3%
	*N*	%
Sex		
Female	3	20%
Male	12	80%
Ethnicity		
Hispanic or Latino	10	66.7%
Not Hispanic or Latino	5	33.3%
Race		
Asian	2	13.3%
Black or African American	1	6.7%
More than one race	1	6.7%
Unknown/not reported	5	33.3%
White	6	40.0%
Stroke type		
Hemorrhagic	5	33.3%
Ischemic	10	66.7%
Stroke laterality		
Left	10	66.7%
Right	5	33.3%
Comorbidities		
Hypertension	13	86.7%
Hyperlipidemia	10	66.7%
Diabetes	6	40.0%
Smoking history	7	50.0%
Current smoker	2	14.3%
Stroke onset symptoms		
Weakness	14	93.3%
Confusion	2	13.3%
Change in sensation	4	26.7%
Difficulty speaking	10	67.7%
Vision changes	2	13.3%

NIHSS, National Institute of Health Stroke Scale; SD, standard deviation.

### Feasibility

Of the 15 participants, four withdrew from the intervention to allow participant or caregiver to return to work due to financial difficulties, or participant illness, and one completed the intervention but was lost to follow-up for assessments. When excluding only the three participants who withdrew within or before week 1 of intervention, the average weekly number of therapy sessions completed was 5.6 (standard deviation [SD] 0.79), 5.6 (SD 0.90), 5.2 (SD 2.19), and 4.9 (SD 1.98) for weeks 2–5, respectively, as detailed in Table 2. Adverse events that were reported during the study participation included falls (*n* = 4), dizziness (*n* = 2), headache (*n* = 1), shoulder pain (*n* = 1), and hospitalization due to dehydration (*n* = 1). Each instance of a fall occurred outside of the study intervention and was considered mild; no injury occurred and medical attention was not required. Reports of dizziness and headache had been occurring on and off since the onset of the stroke; vitals were monitored throughout sessions and no exacerbation of symptoms occurred with study treatment. Shoulder pain was present outside of the study intervention; the intervention was tailored not to exacerbate the reports of pain. A participant experienced dehydration to the extent that he withdrew from the study due to prolonged hospitalization; this occurred within the first week of intervention and was not deemed related to the study intervention.

**Table 2.** Completion of Study Activities

ID	Baseline	Week 1	Week 2	Week 3	Week 4	Post	Final
1	Yes	6	6	5	4	No	No
3	Yes	6	5	4	5	Yes	Yes
4	Yes	6	6	6	6	Yes	No
6	Yes	6	6	6	6	Yes	Yes
7	Yes	4	5	4	2	Yes	Yes
9	Yes	6	6	6	3	No	Yes
10	Yes	2	0	0	0	No	No
13	Yes	6	6	6	6	Yes	Yes
16	Yes	4	3	0	0	Yes	No
18	Yes	5	6	0	4	No	Yes
20	Yes	6	6	5	6	Yes	Yes
21	Yes	6	6	6	6	Yes	No
22	Yes	0	0	0	0	No	No
23	Yes	0	0	0	0	No	No
24	Yes	6	6	4	6	Yes	Yes
	Mean	4.6 (SD 2.20)	4.5 (SD 2.45)	3.5 (SD 2.64)	3.6 (SD 2.56)		
	Without ID 22/23	5.3 (SD 1.25)	5.2 (SD 1.77)	4 (SD 2.46)	4.2 (SD 2.68)		
	Without ID 10/22/23	5.6 (SD 0.79)	5.6 (SD 0.90)	5.2 (SD 2.19)	4.9 (SD 1.98)		

SD, standard deviation.

### Quantitative outcomes

Table 3 provides descriptive characteristics (mean and SD) for each measurement point across outcomes. Model results indicated very strong (PP > 97%) to extreme (PP > 99%) support in favor of change over time across most outcomes, including decreased mRS and PHQ scores and increased MoCA, SF-12 mental, and most SIS subscale scores. Total marginal improvement was 4.178 on the MoCA, −0.731 on the mRS, and −3.606 on the PHQ-8 from baseline to final assessment. Mean change in participants’ SIS score within strength, ADL, mobility, and hand function subtests of the SIS well exceeded the minimally clinically important difference at the postintervention and final assessment when compared with baseline. The two measures demonstrating relatively less evidence for change over time were SF-12 physical and SIS memory and thinking.

**Table 3.** Statistical Analyses

	Descriptive statistics			GLMM results	
Measure	Pretest (week 0) *n* = 15	Post-test (week 6) *n* = 8	Follow-up (week 10) *n* = 8	Total marginal change	Posterior probability (%)
MoCA	19.3 (6.9)	24.6 (5.9)	23.3 (6.3)	4.178	99.9
mRS	2.8 (1.2)	2.1 (1.3)	2 (1.1)	−0.731	99.4
PHQ-8 total	16.2 (4.1)	12.3 (4.1)	12.9 (3.8)	−3.606	99.9
SF-12 physical	37.1 (8.9)	35.6 (8.6)	37.6 (8.0)	0.870	60.1
SF-12 mental	45.6 (11.5)	56 (8.4)	57.1 (12.5)	10.97	99.3
SIS strength	47.5 (31.2)	71.9 (16.4)	67.2 (29.8)	18.61	99.2
SIS memory	71.9 (25.6)	79.5 (19.9)	75 (30.5)	3.72	74.6
SIS emotion	75 (20.5)	81.3 (13.7)	87.2 (14.2)	10.89	96.8
SIS communication	78.1 (21.0)	90.2 (16.9)	91.1 (15.7)	14.98	99.9
SIS ADL	63 (31.4)	82.2 (16.8)	82.5 (21.0)	16.53	99.3
SIS mobility	55 (30.9)	76 (18.7)	77.8 (19.8)	18.86	99.7
SIS hand	48.3 (38.7)	78.1 (29.3)	68.8 (38.5)	20.90	98.4
SIS participation	46.9 (29.2)	62.9 (13.4)	65.2 (26.5)	19.97	99.7
SIS recovery	52.3 (29.7)	71.3 (23.1)	69.4 (27.7)	19.73	99.3

ADL, activity of daily living; GLMM, generalized linear mixed modeling; MoCA, Montreal Cognitive Assessment; mRS, modified Rankin Scale; PHQ-8, Patient Health Questionnaire; SF-12, short form 12; SIS, Stroke Impact Scale.

Analyses supported the influence of comorbid hypertension, hyperlipidemia, and diabetes over time for scores on some outcomes. mRS scores decreased over time for all participants, with a stronger effect for those without hypertension (−16.8%) than with hypertension (−10.1%), for those without hyperlipidemia (−15.9%) than those with hyperlipidemia (−6.0%), and for those with diabetes (−16.1%) than those without diabetes (−8.8%). Analyses supported increased MoCA scores across participants, with a stronger effect for those with hypertension (+15.6%) than those without hypertension (+7.1%) and for those with hyperlipidemia (+17.5%) than those without (+11.1%). PHQ-8 scores decreased more strongly for those without hyperlipidemia (−21.6%) relative to those with hyperlipidemia (−5.8%).

### Qualitative themes

Nine of 15 participants also completed the semi-structured interview. Seventy-eight percent were male participants, and the majority of the individuals were of Hispanic/Latino ethnicity; see Table 4 for demographics of interviewed participants. Six of the interviews were conducted in English, and the rest were completed in Spanish.

**Table 4.** Demographic Characteristics of Semi-Structured Interview Participants

Characteristics	*N* = 9
Age	56 years (mean)
Gender	
Male participants	78% (7)
Ethnicity	
Caucasian	22% (2)
Hispanic/Latino	67% (6)
Asian	11% (1)
Language	
English	67% (6)
Spanish	33% (3)
Insurance (Y/N)	
Yes	44.5% (4)
No	44.5% (4)
Unknown	11% (1)
Caregiver (Y/N)	
Yes	56% (5)
No	44% (4)
Therapy received	
Physical	56% (5)
Occupational	100% (9)
Speech	44% (4)

Two major themes emerged under the facilitators category: 1—“Perceived Access/Delivery” and 2—“Perceived Therapy Advantages” with three subthemes: therapy perceived as “helpful” (subtheme 2a), behavioral therapy perceived as “beneficial” (subtheme 2b), and gaining information and knowledge related to stroke therapies and prevention (subtheme 2c). Under the Barriers category, one major theme emerged: “Needs not met at the telemedicine encounters.” Participants also provided recommendations on how to improve the TR delivery.

### Facilitators

#### Theme 1: Perceived access/delivery

Participants discussed the advantage of having the ability to see both the therapist and health coach from the comfort of their own home. Participant 7 (male, age 52) detailed the advantage of seeing his therapist from home while emphasizing the exercise aspect of the program: “The exercises that were given to me were based on my surroundings so it’s easier for me to go back and do them.” Participants with high level of disability explained that traveling to the health care facilities could be difficult. Some participants were not able to drive themselves or did not have a reliable transportation to get to the doctor due to their geographical location. Participant 20 (male, age 60) stated: “Zoom sessions I think really helped me because I didn’t have to do any traveling and because I’m not really supposed to drive but I didn’t have to bother somebody else to take me somewhere to take to see the therapist.”

In addition, participants explained that receiving the program at home allowed them to feel free to talk to and be honest with the therapist/provider. Participants noted that when they go to the doctor, they can hear patients in other rooms. In addition, a few participants noted that they become anxious at the clinic, so they liked being home for their appointments. Participant 7 (male, age 52) stated: “I think it’ll help people especially if they just had their strokes and they’re … not necessarily nervous but anxious about going out, seeing people or going to places they’re not familiar with.”

#### Theme 2: Perceived therapy advantages

##### Subtheme 2a: TR perceived as “helpful”

In rehabilitative therapy, treatment plans were created based on participants’ specific impairments and goals; visual guides for therapeutic activities were provided. Participant 20 (male, age 60) stated: “She always had pictures that she could show me, this is what I’m trying to get you to do. So that was easy.” Participants also commented on the specific tailored approach to the therapy and shared: “it was full-range occupational therapy so I could get back the movement in my left arm, and I found it very helpful” (Patient 4, female, age 42). Participants also shared their experience regarding the value of the program in the terms of their own ability to be able to continue with a given exercise regimen upon completion of the intervention: “I think the program is going to be very sustainable for me because we made small goals” (Patient 4, female, age 42). She further concluded: “That is what really was the most beneficial part was getting the educational part from y’all as well as from the therapist, the physical therapist and the occupational therapist. That way, if I did not remember something that we discussed in our meeting, I had references to go back to look at to help me stay on task.”

##### Subtheme 2b: Behavioral therapy perceived as “beneficial”

Education regarding stroke risk factors control and secondary prevention materials was offered to participants to educate them on what can be done to protect them from having a recurrent stroke. Participant 20 (male, age 60) stated: “It was beneficial in that in a sense cemented some of what I already knew because these things are discussed especially when signing up for medical insurance and the company you work for is pushing for healthy choices.”

Participant 21 (male, age 64) along with his caregiver also added to it: “Now we’re aware of what we’re eating wrong and the portions.”

Coaching techniques, such as breathing exercises and meditation, taught participants to have more control over their emotions. It was understood that if they felt better emotionally, then they could do better with their long-term rehabilitation plan. Patient 4 (female, age 42) best described it as: “I learned how to do the breathing exercises, thinking exercises, meditation exercises, so as not to react or how to react calmly to situations instead of crying or getting all upset because you felt better emotionally you could carry on with the exercise, you know, it helped you to do that.” Participant 24 (male, age 32) shared that he needed more time to notice the results of behavioral interventions. The participant stated: “It was a little difficult at first but as I kept working on it. I wanna say, maybe by the second week is when I’ve been noticing that I’ve been getting a little bit better than the beginning. I still have difficulty … but I’ve been trying to learn to slow down my breathing … to give to give my brain a little bit more time to get the words out better.”

A commonly cited motive expressed by participants regarding benefits of behavioral therapy was their ability to utilize the goal-oriented behavior change techniques. Patients shared their stroke risk factors with the coach, were introduced to the goal-setting concept, thinking of how to develop action plans focused on specific behavior, and the ways to adjust this behavior. Patient 4 (female, age 42) stated that she found goal setting “very attainable.” She felt that she knew she really needed to push herself because she knew she could do better. She stated: “now I have the tools to set the goals.” Patient 20 (male, age 60) also commented: “… before this therapy I was just winging it.”

##### Subtheme 2c: Gaining information and knowledge related to stroke therapies and prevention

The information exchange between participants, therapists, and coaches was described as an important part of the program intervention. Evidence-based materials and tailored education focused on participants’ specific needs, allowing them to create attainable goals. Participant 20 (male, age 60) stated: “These sessions gave me actual, specific goals that I had to try to achieve or work to achieve to get to where I am at now, I have the tools to … set the goals.” In addition, participants gained new perspective on how they can move forward past the program and work on their own to adopt new exercise skills and use goal-oriented lifestyle changes. Patient 4 (female, age 42) commented: “That is what really was the most beneficial part was getting the educational part from y’all as well as from the therapist, the physical therapist and the occupational therapist.”

### Category: Barriers

#### Theme 1: Unmet needs during the telemedicine visits

Though most patients felt there weren’t really any barriers to the program, a few felt that not being in the same room with the clinician was a disadvantage. They wanted the therapist next to them giving them specific instructions. They felt having the therapist next to them would help them push harder. This is best described by Patient 4 (female, age 42): “The hardest part was that they weren’t right next to me, they never pushed me harder.” One participant commented that he felt that they should be seen in person for the first visit and follow-up with the telemedicine visit: “I don’t know I’ve never been to actual to rehabilitation like in person but I know that I’d probably be better because I’d probably be able to hear questions better that way because from … the laptop I know sometimes it makes the questions difficult when I have hard time hearing it …” (Patient 21, male, age 64). Another participant added: “I don’t think that there was anything that was strongly difficult. I think that it would have been more beneficial in person, showing me how to properly use measuring cups, measuring tools. Portion sizes or how to weigh food …” (Participant 24, male, age 32).

### Final recommendations

Most participants were satisfied and did not recommend changes in terms of the content or the program delivery method. Several participants felt that the program should be extended to allow for more frequent and longer visits: “The only thing would be either more time or more days because your program is very good” (Patient 13, male, age 75). Patient 20 (male, age 60) stated: “It would have been nice for more time.” In addition, patients perceived the TR stroke program as an important poststroke health care delivery model. Participant 7 (male, age 52) said: “I think it’s an excellent program. It’ll help, I think it’ll help people especially if they just had their strokes and they’re … not necessarily nervous but anxious about going out … I think it is a lot better for those people unless the people are just, I don’t know, maybe not willing to put in the work.”

## Discussion

VAST-rehab 2 explored an intensive TR intervention combined with SMS for virtual postacute stroke care and obtained qualitative feedback on TR facilitators and barriers. Compared with prior TR publications, our study is unique by providing completely remote assessment, rehabilitation therapy, and SMS intervention without reliance on outside technology, such as virtual reality or hybrid TR studies. No in-person study activities were conducted. The exclusion of complicated technology can allow for a more personalized TR experience while still facilitating improvement in outcomes.

This study sample was predominantly young, male, and Latino with low education levels, mild strokes, multiple comorbidities, and located in two disparate locations in Texas. Studies have found that those who are younger and with a lower National Institute of Health Stroke Scale are more likely to discharge home than to inpatient rehabilitation after acute stroke hospitalization^35^; however, up to 59% will not see a rehabilitation therapist within 30 days of discharge.^36^ These numbers are staggering considering the narrow neuroplasticity window open in this time frame,^37–39^ as well as a reduced likelihood of hospital readmission for those that receive therapy within that time frame.^36^ While barriers to rehabilitation utilization following acute hospitalization are variable, the use of TR can facilitate rehabilitation within a very critical window of recovery, minimizing barriers related to transportation and geography.^40^ Furthermore, behavioral therapy was perceived as beneficial, and SMS education was recognized as useful to gain information and knowledge related to stroke therapies and prevention. Similar findings were reported by Knepley et al.^41^ and Kimmel et al.^40^

Of the participants that completed the study, attendance was high, averaging around five sessions each week or an 83% attendance rate. This level of attendance demonstrates feasibility for an intensive and high-dosage TR program for stroke survivors after discharge from acute hospitalization. Further demonstrating feasibility, the majority of participants recommended the therapy program actually be longer in duration. Rehabilitation utilization after stroke is woefully low, with some studies suggesting that stroke survivors residing in the community do not receive comparable care as those with other neurological diagnoses.^42,43^ The results of this study demonstrate that TR provides an opportunity to increase rehabilitation utilization for stroke survivors within their own homes.

Throughout the study, no adverse events occurred that were related to the study intervention. Based on this information and the qualitative participants’ perspectives, TR and SMS can be viewed as a safe and feasible option for postacute rehabilitation after stroke for survivors with mild to moderate impairment. The most frequent adverse event that occurred outside of study activities was falls. Falls after stroke are very common and are most likely to occur within the first 2 months after discharging home.^44,45^ Regular interaction with rehabilitation therapists and health coaches through TR can provide fall prevention and postfall debriefing. Therapists and health coaches interacting with stroke survivors within the home can explore the interlinking factors that play a role in the fall, diving deeper into the activity, participation, body function, environmental, and personal factors involved. Using postfall debriefing within the natural environment of the home where the fall occurred may lead to better learning and retention of safety techniques. In this study, rehabilitation therapists led the participants that reported falls through this process as a teachable moment for future fall prevention and overall fall safety.

Most quantitative outcomes improved postintervention and continued to improve or were maintained at the final assessment.^46^ These results indicate that the TR intervention improved function and reduced disability. This aligns with the changes on the mRS, with average mRS scores decreasing postintervention and continuing at 6 weeks follow-up, as well as participant reports that the program was beneficial for their recovery. From the individual participant level, 60% of participants demonstrated a reduction in their mRS score by at least 1 level.

While the minimally clinically important differences (MCIDs) are not readily available for the SIS subtests of memory/thinking, emotions, communication, and role participation, average scores between time points increased in all areas except for memory and thinking. However, the perceived lack of change in memory and thinking that is reported on the SIS does not align with the results of the MoCA. Seventy percent of the participants moved to a lower cognitive impairment severity level on the MoCA at follow-up, with 7% demonstrating no cognitive impairment at baseline, 64% at post-intervention, and 50% at final follow-up.^47^ Sixty percent of participants scored within the mild cognitive range at baseline, reduced to 25% at post-intervention, and 38% at final follow-up. These results indicate that executive functioning did improve for the majority of participants.

TR utilization was prevalent during the COVID-19 pandemic; however, continued frequency of its use is unclear. Despite this, the Center for Medicare and Medicaid Services continues to review and approve the use of rehabilitation therapy billing codes for telehealth services.^48^ In the state of Texas, rehabilitation therapists’ licenses allow for use of clinical judgment in regard to which aspects of an evaluation and intervention can be completed remotely.^49–51^ The state of Texas has one of the highest rural populations in the United States; 71% of Texas’s rural counties have 0 outpatient rehabilitation clinics.^52^ There is a great opportunity for TR to be utilized within clinical settings to provide specialized neurorehabilitation to underserved populations in rural areas.

### Limitations

Due to funding and time limitations, the sample size of this study was low, which limits generalizability outside of the participants, as well as the ability to incorporate a control group into the sample size. Confirmation bias may also be a limitation within the study, as participants may have expected improvements following TR. The findings from GLMM analyses should be considered preliminary and hypothesis-generating. In regard to the goal attainment, low sample sizes limited precision for analyses, thus these were not included in analyses. Future research is being planned and should include large sample sizes to analyze treatment effects.

Attrition is always a limitation in research, especially in small studies. However, within this study, one should note that the attrition rate is similar to that of traditional outpatient therapies.^53^ Another challenge that limited participation was the use of technology. Some of the potential participants had access to the technology (smartphones, laptops, tablets) but were unfamiliar with video call platforms and, in turn, unable to participate in a TR intervention. This limitation and mitigation strategies should be further explored with consideration of a minimal level of digital competence required within the study population for participation in TR. Larger studies will allow for further analyses of correlation among social determinants of health and attrition.

## Conclusion

VAST-rehab 2 explored a fully remote TR assessment and intervention combined with SMS for feasibility, impact on functional and disability outcomes, and goal attainment. This study proved to be a safe and attainable option for underserved populations of stroke survivors, demonstrating high attendance, improved outcomes, no intervention-related adverse events, and positive reception from participants. Participants demonstrated statistically significant improvements in disability on the mRS, quality of life on the PHQ-8, and cognitive outcomes on the MoCA. Combination of TR and SMS should be further explored in larger randomized controlled trials to explore treatment effects across stroke survivors with assorted comorbidities and variable levels of impairment. We are continuing our work in TR use with stroke survivors throughout the state of Texas through a gap analysis, identifying supports and barriers to implementation. In addition, new studies are exploring/addressing decision support systems,^54^ which will also be helpful in future planning for the value of TR testing in larger communities. Concurrently, we are developing training resources that will be tested through implementation science within the TR community in Texas.

## Abbreviations Used

ADLs: Activities of daily living
BF: Bayes factor
CMS: Center for Medicare and Medicaid Services
CrI: Credible intervals
DALYs: Disability-adjusted life year
GLMM: Generalized linear mixed modeling
HIPAA: Health Insurance Portability and Accountability Act
MCID: Minimally clinically important differences
MoCA: Montreal Cognitive Assessment
mRS: Modified Rankin Scale
NIHSS: National Institute of Health Stroke Scale
PHQ8: Patient Health Questionnaire
PP: Posterior probability
SD: Standard deviation
SF-12: Short Form 12
SIS: Stroke Impact Scale
SMS: Self-management support
TR: Telerehabilitation
VAST-rehab2: Virtually assisted home rehabilitation after acute stroke - 2
